# Screening a Natural Product-Based Library against Kinetoplastid Parasites

**DOI:** 10.3390/molecules22101715

**Published:** 2017-10-12

**Authors:** Bilal Zulfiqar, Amy J. Jones, Melissa L. Sykes, Todd B. Shelper, Rohan A. Davis, Vicky M. Avery

**Affiliations:** 1 Discovery Biology, Griffith Institute for Drug Discovery, Griffith University, Nathan, Queensland 4111, Australia; bilal.zulfiqar@griffithuni.edu.au (B.Z.); a.jones@griffith.edu.au (A.J.J.); m.sykes@griffith.edu.au (M.L.S.); t.shelper@griffith.edu.au (T.B.S.); 2 Natural Product Chemistry, Griffith Institute for Drug Discovery, Griffith University, Nathan, Queensland 4111, Australia; r.davis@griffith.edu.au

**Keywords:** natural products, kinetoplastids, neglected tropical disease, drug discovery, leishmaniasis, human African trypanosomiasis, Chagas disease

## Abstract

Kinetoplastid parasites cause vector-borne parasitic diseases including leishmaniasis, human African trypanosomiasis (HAT) and Chagas disease. These Neglected Tropical Diseases (NTDs) impact on some of the world’s lowest socioeconomic communities. Current treatments for these diseases cause severe toxicity and have limited efficacy, highlighting the need to identify new treatments. In this study, the Davis open access natural product-based library was screened against kinetoplastids (*Leishmania donovani* DD8, *Trypanosoma brucei brucei* and *Trypanosoma cruzi*) using phenotypic assays. The aim of this study was to identify hit compounds, with a focus on improved efficacy, selectivity and potential to target several kinetoplastid parasites. The IC_50_ values of the natural products were obtained for *L. donovani* DD8, *T. b. brucei* and *T. cruzi* in addition to cytotoxicity against the mammalian cell lines, HEK-293, 3T3 and THP-1 cell lines were determined to ascertain parasite selectivity. Thirty-one compounds were identified with IC_50_ values of ≤10 µM against the kinetoplastid parasites tested. Lissoclinotoxin E (**1**) was the only compound identified with activity across all three investigated parasites, exhibiting IC_50_ values <5 µM. In this study, natural products with the potential to be new chemical starting points for drug discovery efforts for kinetoplastid diseases were identified.

## 1. Introduction

Trypanosomatida is a group of kinetoplastid protozoa differentiated into the genus *Leishmania* and *Trypanosoma*. Parasites belonging to the genus *Leishmania* are the causative agents of leishmaniasis*,* while *Trypanosoma brucei gambiense* and *Trypanosoma brucei rhodesiense* are the causative agents of human African trypanosomiasis (HAT). The other parasite in the genus *Trypanosoma* is *Trypanosoma cruzi*, which is responsible for Chagas disease. Around 20 million individuals are infected with kinetoplastid pathogens worldwide leading to 95,000 deaths per year [1]. For Chagas disease and HAT the primary areas of transmission are Latin America and sub-Saharan Africa, respectively [2]. Leishmaniasis is endemic to 98 countries around the globe [3].

The vector for leishmaniasis is the *Phlebotomus* sand-fly in the Old World and the *Lutzomyia* sand-fly in the New World [4]; while *T. b. gambiense* and *T. b. rhodesiense* are transmitted by the bite of a tsetse fly [5]. *T. cruzi* is primarily transmitted by triatomine bugs to humans through contact with bug faeces or urine at the site of a bite [6].

The complex multiple host life cycles of these kinetoplastids provides a challenge for drug discovery efforts thus it is preferable that where possible, drugs are able to target several of these life cycle stages, species and in addition, stages of infection. The life cycle of the *Leishmania* parasite constitutes two key forms; the promastigotes which reside within the gut of the sand fly and the intracellular amastigotes within the mammalian host macrophages [7]. Based on clinical manifestations, leishmaniasis can be differentiated into cutaneous, mucocutaneous and visceral leishmaniasis [8]. *T. b. gambiense* and *T. b. rhodesiense* exist as extracellular forms in the tsetse fly as procyclic and metacyclic trypomastigotes. Once injected into the host, they transform into bloodstream trypomastigotes. HAT progresses in two stages: a hemolymphatic stage and a central nervous system (CNS) stage [9]. The *T. cruzi* life cycle involves three distinct stages; epimastigotes reside within the gut of the triatomine bug where they divide and differentiate into infective metacyclic trypomastigotes in the hindgut. Like leishmaniasis, inside the host they transform into intracellular amastigotes within the infected cell. In Chagas disease; early (acute) stage of the disease may be asymptomatic or display only mild symptoms, while a latter, chronic stage results in cardiac disorders (30%) or digestive disorders (10%), due to the parasite residing in the tissue, or due to associated inflammation in tissue free of the parasite [10]. Kinetoplastid diseases are extremely debilitating and can be fatal without treatment [11].

Existing treatments for leishmaniasis such as miltefosine and amphotericin B deoxycholate [12] have limitations and toxicities associated with them. Current treatments for HAT including pentamidine and eflornithine have variable efficacy at the different stages of the disease and against the different subspecies of *T. brucei*, and are fraught with severe side effects [13]. For Chagas disease, treatment with the drugs benznidazole and nifurtimox is most effective in the early stages of the disease, but efficacy diminishes with duration of infection [14]. Collectively, the therapies available for kinetoplastid diseases are inadequate, lack efficacy and possess extensive toxicity. Additionally, there is evidence of emerging or potential resistance in leishmaniasis [15], HAT [16], Chagas disease [17] and access to the drugs in remote areas is limited [18,19,20]. Despite the new drug leads currently in clinical trials for leishmaniasis [21,22,23], HAT [24,25,26] and Chagas disease [27], there exists a high attrition rate [28,29] and new molecules with novel mechanisms of action are required.

Natural products have traditionally been used to treat parasitic diseases, primarily through ethnopharmacology approaches [30]. More recent efforts to elucidate the structural and biological properties of the chemical entities within complex anti-parasitic natural product extracts have identified molecules with significant potential for treating NTD’s. For example, recently the antiprotozoal activity of the isolated alkamide dodeca-2*E*,4*E*-dienoic acid 4-hydroxy-2-phenyl-ethylamide from *Anacyclus pyrethrum* roots has been reported against *L. donovani*, *T. b. rhodesiense*, *T. cruzi* and the NF54 strain of *Plasmodium falciparum* with an IC_50_ of 4.19 ± 1.64, 2.26 ± 0.18, 1.88 and 3.18 ± 0.20 µM, respectively [31]. Australia has a high level of biodiversity providing an exceptional resource for natural product drug discovery. We have previously reported the identification and biological profiling of compounds originating from plants [32], marine invertebrates [33,34,35] and fungi [36], which possess anti-parasitic activity.

In our continuing search for new anti-parasitic compounds from nature, we report here the identification of several bioactive molecules with activity across multiple life cycle stages of three kinetoplastids, *L. donovani* DD8 (visceral leishmaniasis: intracellular amastigotes and extracellular promastigotes residing in the gut of sandfly)*, T. b. brucei* (a surrogate species for HAT: bloodstream trypomastigotes) and *T. cruzi* (Chagas disease: intracellular amastigotes). This is the first evaluation of the unique Davis open access natural product-based library against kinetoplastids which has resulted in the identification of several compounds with novel anti-kinetoplastid activities.

## 2. Results

### 2.1. Screening Campaigns and Hit Identification

#### 2.1.1. *L. donovani DD8* Promastigote and Intracellular Amastigote Screening

**Figure 1:**
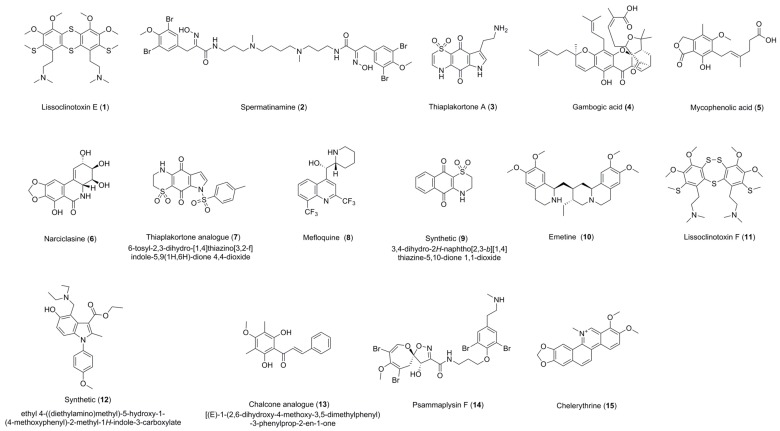
Structures of compounds **1**–**15**.

Twenty nine compounds exhibited >70% activity at 16.7 µM against promastigotes with a hit rate of 6.14%. Twelve compounds were active against intracellular amastigotes, with ≥70% inhibition at 20 µM. Of these, 6 compounds showed cytotoxicity against THP-1 cells at 20 µM. Following retest, five compounds exhibited IC_50_ values of <10 µM against *L. donovani* DD8 intracellular amastigotes. These five compounds demonstrated comparable activity against both forms (namely promastigote and amastigote), whereas compound (**1**) (Figure 1) exhibited more potent activity against the promastigotes (IC_50_: 0.73 ± 0.16 µM) than the amastigotes (IC_50_: 4.41 ± 0.24 µM). Of these compounds, compound (**13**) displayed good selectivity of ~12 against the parasite in relation to HEK-293 cells. The Z’ of the intracellular amastigote and promastigote viability assays for primary screen and retest indicated high reproducibility with values of (0.75, 0.72) and (0.91, 0.90), respectively. A Z’ for assays using THP-1 and HEK-293 cells were calculated to be (0.70, 0.69) and (0.78, 0.72), respectively.

The IC_50_ values for the reference drugs amphotericin B and miltefosine were 0.12 ± 0.01 µM, 0.20 ± 0.02 µM and 3.48 ± 0.26 µM, 2.54 ± 0.57 µM for the promastigote viability and intracellular amastigote assays, respectively (Table 1). The IC_50_ value for miltefosine was 2.54 ± 0.57 µM in the amastigote assay, which is consistent with data previously reported in the literature (3.1 ± 2.3 µM) [37].

#### 2.1.2. *T. b. brucei* Screening

A *T. b. brucei* resazurin viability assay [38] identified 28 compounds with a cut-off of ≥50% activity at 5 µM, indicating a hit rate of 5.93%. Whilst all compounds confirmed activity at the primary screening dose (5 µM), accurate IC_50_ values could only be determined for 21 compounds. These compounds exhibited IC_50_ values between 0.05 and 4.84 µM against *T. b. brucei*, with two compounds showing good selectivity (~18 fold) against HEK-293 cells.

The three reference drugs/compounds used as controls for the *T. b. brucei* assay were pentamidine, diminazene and puromycin. The IC_50_ values for pentamidine, diminazene and puromycin were found to be 2.86 ± 1.46 nM, 40.51 ± 12.76 nM and 38.96 ± 6.09 nM, respectively (Table 1) which correlate with previously reported values [39].

#### 2.1.3*. T. cruzi* Intracellular Amastigote Screening

Using a *T. cruzi* intracellular amastigote image-based assay [40], 15 compounds with a cut off of ≥50% activity at a concentration of 10 µM were identified, thus a hit rate of 3.17%. In addition to determining the IC_50_ value against *T. cruzi* and the 3T3 host cells, compounds were also tested in a HEK-293 assay to determine the activity against a dividing human cell line. The Z’ values obtained for the intracellular amastigote, 3T3 host cell and HEK-293 retest assays were 0.63 ± 0.06, 0.71 ± 0.01 and 0.77 ± 0.03, respectively.

The IC_50_ values for the reference drugs benznidazole and nifurtimox, as well as puromycin were 3.36 ± 1.52 µM, 0.62 ± 0.10 µM and 1.65 ± 0.35 µM, respectively as shown in Table 1. These correlate with previously published values [40].

#### 2.1.4. Common Activity Against Kinetoplastids

**Figure 2:**
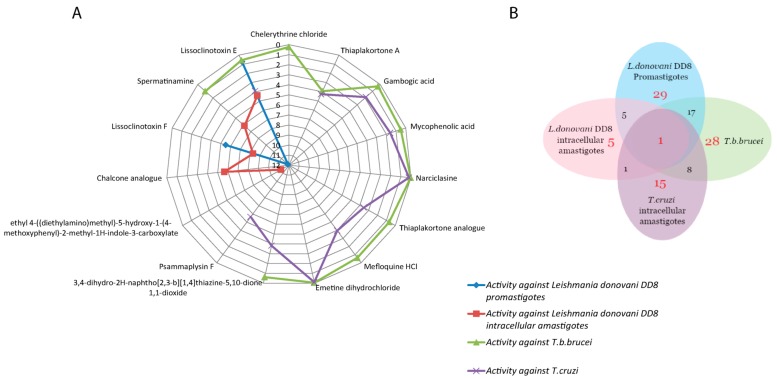
(**A**) Spider plot displaying the IC_50_ values of compounds for *L. donovani* DD8 promastigotes, *L. donovani* DD8 intracellular amastigotes, *T. b. brucei* and *T. cruzi*; (**B**) Venn diagram for overlapping active hits identified against *L. donovani* DD8 promastigotes, *L. donovani* DD8 intracellular amastigotes, *T. b. brucei* and *T. cruzi*.

The activity of compounds against multiple kinetoplastid parasites is described in Table 2. Compounds lissoclinotoxin E (**1**) and spermatinamine (**2**) demonstrated activity against both *L. donovani* DD8 forms and *T. b. brucei*. Seventeen compounds which were active against *L. donovani* DD8 promastigotes were also active on *T. b. brucei*, with eight compounds showing activity on both *T. b. brucei* and *T. cruzi* (Figure 2A). Compound (**1**) was active on all three parasites with an IC_50_ value of <5 µM, however only showed moderate selectivity for *T. b. brucei* (Figure 2B).

## 3. Discussion

The drug discovery pipeline for neglected trypanosomatid diseases remains sparse. Drug discovery has had limited success in translating potential drug candidates into viable therapies for HAT, leishmaniasis and Chagas disease. Natural products serve as an attractive alternative source of chemical starting points for drug discovery against kinetoplastid parasites [47,48]. The objective of this work was to identify actives from parallel screening of 472 natural product-derived compounds against the parasites responsible for leishmaniasis, HAT and Chagas disease.

Kinetoplastid pathogens share much of their cellular and molecular biology even though they cause clinically distinctive diseases and are trasmitted by different insect vectors [49]. The parasites in these studies share common molecular targets including trypanothione reductase, pteridine reductase and cysteine protease enzymes, which have been proposed for target-based screening [12,13,14]. Inhibitors have been designed in the past for these enzymes as anti-leishmanials [50,51,52] and anti-trypanosomals [53,54,55]. Fexinidazole is the most advanced oral candidate under development for Chagas disease and HAT, currently in the Phase II and Phase III clicical trials, respectively [56]. This lead compound has also shown potent activity against *L. donovani* in vitro and in vivo in a visceral leishmaniasis mouse model, illustrating the significant value of screening against multiple kinetoplastids [57].

A target candidate profile (TCP) [18] has been established by Drugs for Neglected Diseases initiative (DND*i*) to classify a compound as an active hit against *Leishmania*, *T. b. brucei* and *T. cruzi*. We have used their criteria in terms of efficacy in vitro with an IC_50_ < 10 µM as the classification of a potential anti-kinetoplastid hit. For *L. donovani* DD8, less priority has been given to compounds only active on promastigotes alone in comparison to compounds active only on intracellular amastigotes, as intracellular amastigotes represent the pathophysiological relevant form of the disease [37]. 

Using the SMILE string of active compounds, which was obtained using ChemSpider (http://www.chemspider.com/), previously reported biological activities of compounds were identified using SciFinder (http://www.cas.org/products/scifinder) and ChEMBL (https://www.ebi.ac.uk/chembl/).

**Figure 3:**
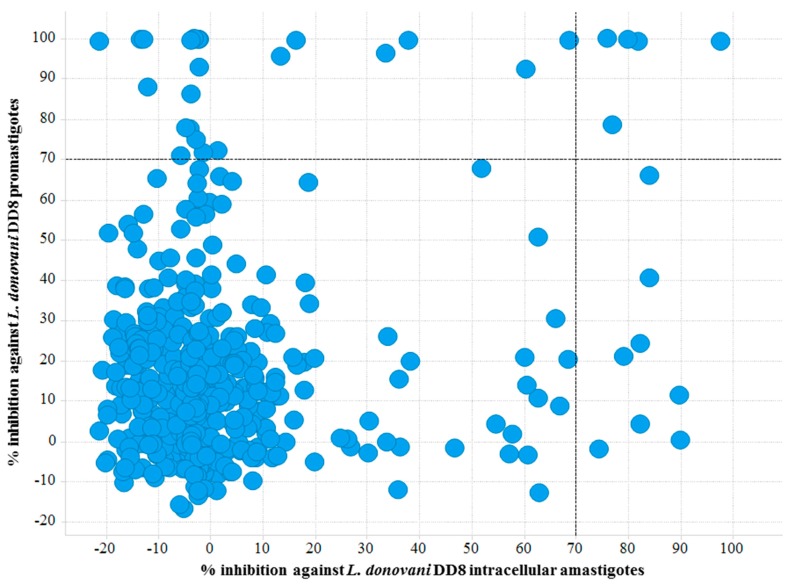
Scatter plot of active hits against *L. donovani* DD8 promastigotes and *L. donovani* DD8 intracellular amastigotes. Right top quadrant exhibiting compounds found to be active on both *L. donovani* DD8 promastigotes and *L. donovani* DD8 intracellular amastigotes.

Twenty-nine compounds exhibiting >70% activity at 16.7 µM in the *L. donovani* promastigote viability assay were identified, giving a hit rate of 6.14% (Figure 3) compared to the outcomes from the amastigote screening which identified 6 compounds with a hit rate of 1.27%. We predict that the differences observed in these hit rates are due to several factors including the parasite environment (intracellular vs extracellular); compound permeability and potential effects of the environmental pH on the compound within the parasitophorous vacuole. A similar high hit rate (5.93%) was observed for *T. b. brucei* which is also an extracellular parasite. Further evidence supports this finding, as 17 compounds were mutually active on *L. donovani* DD8 promastigotes and *T. b. brucei* (Figure 1B).

Of the 472 compounds screened, 8 compounds showed activity against both *T. b. brucei* (extracellular) and *T. cruzi* intracellular amastigotes (Figure 1B). As the biochemical similarity between *T. b. brucei and T. cruzi* is much greater than that for *Leishmania* [58], this may explain why the number of mutual hits between them is much higher than between *T. b. brucei* and intracellular *Leishmania,* and *T. cruzi* and intracellular *Leishmania.*

Lissoclinotoxin E (**1**) was the only compound that was pan-active against all three parasites tested with an IC_50_ value of <5 µM. Lissoclinotoxin E was identified as one of two new dimeric alkaloids [the other is lissoclinotoxin F (**11**)] which were both initially purified from a didemnid ascidian [59]. Lissoclinotoxin E exhibited an IC_50_ value of 0.57 ± 0.20 µM and 3.92 ± 0.38 µM against *T. b. brucei and T. cruzi,* respectively. The antileishmanial activities for compound **1** on promastigotes and intracellular amastigotes were 0.72 ± 0.16 and 4.41 ± 0.24 µM, respectively. Compound **1** exhibited high selectivity for *T. b. brucei* in relation to HEK 293 cells. The selectivity for HEK-293 and 3T3 cells needs to be confirmed at higher doses for *L. donovani* DD8 and *T. cruzi*.

The other dimeric alkaloid, lissoclinotoxin F (**11**), also showed activity against both forms of the *Leishmania* parasite (5.51 ± 0.30 and 8.31 ± 0.67 µM, respectively), albeit lower. This is novel activity reported for these compounds against kinetoplastid parasites. Previously, both these compounds have shown interesting activity against PTEN-deficient (PTEN-/-) cell line indicating antitumor activity [59]. This class of compound has shown prominent activity against all three parasites and it could be of interest to further explore the antiprotozoal potential of this class. Analogues can be synthesized which may address any potential cytotoxicity issues.

Spermatinamine (**2**), isloated from the sponge *Pseudoceratina* sp. displayed activity on both forms of *L. donovani* DD8 and *T. b. brucei.* This compound displayed IC_50_ values of 6.15 ± 0.05, 11.87 ± 0.56 and 1.00 ± 0.26 µM against *L. donovani* DD8 intracellular amastigotes, promastigotes and *T. b. brucei* parasites, respectively. Compound **2** exhibited moderate selectivity for *T. b. brucei* in relation to HEK 293 cells with no selectivity for *L. donovani* DD8 intracellular amastigotes and promastigotes. Spermatinamine was originally discovered as a inhibitor of isoprenylcysteine carboxyl methyltransferase (ICMT) [60]. ICMT is a potential target for cancer therapeutics as this enzyme catalyses the final step in post translational modification, where carboxyl methylation of oncogenic proteins takes place. Spematinamine has also been shown to have antibacterial activity against Gram-negative bacteria [61] as well as having antimalarial activity (IC_50_: 0.23 µM) against *P. falciparum* (3D7) parasites [62]. Therefore, this may also be the target of this compound in *T. b. brucei* parasites and warrants further investigation, as this parasite also shares a protein carboxyl methyltransferase [63]. This is the first time that the activity of these compounds has been reported against these parasites and it would be beneficial to identify more kinetoplastid-selective compounds from this class.

On the basis of the information currently available, it seems fair to suggest that thiaplakortone A (**3**) and thiaplakortone analogue **7** have novel activity against both *T. b. brucei* and *T. cruzi* parasites. In addition, gambogic acid (**4**), mycophenolic acid (**5**), narciclasine (**6**), 3,4-dihydro-2*H*-naphtho[2,3-*b*][1,4]thiazine-5,10-dione 1,1-dioxide (**9**) and psammaplysin F (**14**) have novel activity against *T. cruzi* parasites.

Thiaplakortone A (**3**) is a thiazine-derived alkaloid, initially isolated from the marine sponge *Plakortis lita* [35]. This compound was active against *T. b. brucei* and *T. cruzi* with IC_50_ values of 3.94 ± 0.71 and 4.26 ± 0.65 µM, respectively. Compound **3** exhibited no selectivity for *T. b. brucei* and *T. cruzi.* Interestingly, a truncated synthetic thiaplakortone analogue **7**, 6-tosyl-2,3-dihydro-[1,4]thiazino[3,2-*f*]indole-5,9(1*H*,6*H*)-dione 4,4-dioxide [64], has also been shown to be active against *T. b. brucei* and *T. cruzi* (IC_50_ of 0.68 ± 0.01 and 3.55 ± 0.38 µM, respectively). Compound **7** has exhibited better selectivity for *T. b. brucei* and *T. cruzi* than compound **3**, indicating other analogues should be explored to identify selective compounds. Compound **3** was previously reported to have potent activity against both chloroquine-sensitive (3D7) and multi-drug resistant (Dd2) *P. falciparum* parasites (IC_50_ of 51, and 6.6 nM, respectively) [35]. Compound **7** has also been tested for activity against 3D7 and Dd2 strains of *P. falciparum* with reported IC_50_ values of 546 ± 119 and 509 ± 30.9 nM [64]. The significant difference in plasmodial activity between **3** and **7** may be due to the different thiazine regiochemistry and the lack of an ethylamine chain in **7**, compared to the natural product **3** ordifferences in the assay formats used. In vivo testing of thiaplakortone A in a mouse model of malaria infection did not suppress parasitemia with oral administration, possibly due to associated poor bioavailability [65].

Gambogic acid (**4**) is a xanthonoid isolated from the gamboge resin of the *Garcinia hanburyi* tree [66] which was demonstrated to have anti-trypanosomal activity against *T. b. brucei* and *T. cruzi* (IC_50_ of 0.27 ± 0.04 and 1.87 ± 0.07 µM, respectively). Compound **4** exhibits moderate selectivity for *T. b. brucei* in relation to HEK-293 cells. *T. b. brucei* activity for compound **4** has been previously reported [67] indicating similar values to those obtained for *T. b. brucei* in this study. The activity in the *T. cruzi* assay is indicative of host-cell damage as this compound is not selective and has also been shown to be cytotoxic, inducing apoptosis in Jurkat cells through caspase 3 activation and mitochondrial destabilization [68].

Mycophenolic acid (**5**) is classified as an inosine 5'-monophosphate dehydrogenase inhibitor and the anti-trypanosomal effects of this compound have been identified against *T. b. brucei* [69] in addition to many other protozoan parasites [70,71]. We observed activity against *T. b. brucei* (IC_50_ of 0.51 ± 0.10 µM) which correlates with the previously reported IC_50_ value [72]. An IC_50_ value of 1.59 ± 0.03 µM was demonstrated in the *T. cruzi* assay and showed no selectivity, thus effecting the host cell. To the best of our knowledge this compound has not been tested against *T. cruzi* previously.

The natural compound narciclasine (**6**) was first isolated from daffodils, which belong to the *Amaryllidaceae* plant family [73]. This compound was active against *T. b. brucei* and *T. cruzi* (IC_50_ values of 0.03 ± 0.01 and 0.20 ± 0.01 µM, respectively) and like gambogic acid, has been previously reported with activity against *T. b. brucei* [67] but not for *T. cruzi*. Compound **6** has no selectivity for *T. b. brucei,* or *T. cruzi* being equally toxic against the mammalian cells. The mechanism of action of this compound suggests that it inhibits protein synthesis by blocking peptide bond formation in *Saccharomyces cerevisiae* yeast [74]. The compound has anti-viral activity against yellow fever, Japanese encephalitis and dengue fever but due to off target toxicity it has not been tested in in vivo models [75]. Whether a similar mechanism of action is the means by which this compound exerts the effects we observed has not been verified, however as the activity is non-specific, pursuing this compound further is of limited value.

Compounds **9** and **14** showed interesting activity against *T. cruzi* intracellular amastigotes with IC_50_ values of 3.82 ± 0.61 and 5.64 ± 0.76 µM, respectively. Compound **9** is a synthetic compound, which exhibits no selectivity for *T. cruzi*. Compound **14**, a bromotyrosine alkaloid named psammaplysin F was isolated from the marine sponge, *Hyatella* sp [76]. Although IC_50_ values could not be determined for the host cells or HEK-293 cells for this compound, there was similar activity against the mammalian cell at the concentrations eliciting activity for *T. cruzi*. This compound has been shown to exhibit antimalarial activity against *P. falciparum* strains (3D7 and Dd2) with an IC_50_ value of 0.87 and 1.4 µM, respectively [76].

Compounds **12** (ethyl 4-((diethylamino)methyl)-5-hydroxy-1-(4-methoxyphenyl)-2-methyl-1*H*-indole-3-carboxylate) and **13** [(*E*)-1-(2,6-dihydroxy-4-methoxy-3,5-dimethylphenyl)-3-phenylprop-2-en-1-one] showed activity against both intracellular and extracellular forms of *L. donovani* DD8. Compound **12** and **13** displayed IC_50_ values of 11.09 ± 0.311 and 5.65 ± 0.26 µM against *L. donovani* DD8 intracellular amastigotes. Both of these compounds showed no significant activity against either *T. b. brucei* or *T. cruzi*. Compound **13** has shown high selectivity for *L. donovani* DD8 intracellular amastigotes in relation to HEK-293 cells. Compound **13** was previously reported to have moderate activity against both *L. donovani* (MHOM/SD/62/1S-CL2D) axenic amastigotes and *T. b. brucei* variant 221 with IC_50_ values of 16.75 ± 4.35 and 21.11 ± 3.68 µM, respectively [77]. Compound **13** also caused a 96 ± 2% reduction in infection of *L. mexicana* intracellular amastigotes at 41.89 µM [77]. Previously, it has been reported that compound activity varies based on the geographical location of the species and strains of kinetoplastid parasites [78]. An example in this context is parmomycin, which has negligible activity against *L. donovani* DD8 when compared to other strains [79]. This may explain the difference of activity observed for compound **13** for *Leishmania* species; however, differences in assays may also play a role. Compound **13** is a promising hit as it fulfils the TCP criteria set by DND*i* for anti-leishmanial drug discovery. It will be interesting to further explore this compound by synthesising analogues and assessing structure activity relationships.

A small percentage (5%) of the library is comprised of commercial or synthetic compounds inspired by natural products. Mefloquine HCl (**8**), emetine dihydrochloride (**10**), and chelerythrine chloride (**15**) which belong to this sub-group were shown to have activity against *L. donovani* DD8, *T. b. brucei* and *T. cruzi* serving as internal controls. The 50% inhibitory concentration observed for mefloquine HCl (**8**) was 0.62 ± 0.06 and 3.96 ± 0.58 µM for *T. b. brucei* and *T. cruzi*, respectively, with moderate selectivity for *T. b. brucei*. Mefloquine has previously been shown to have activity against *T. b. brucei* in vitro and in vivo [80] and has a reported EC_50_ of 6.1 µM against *T. cruzi*, with a SI of 2 to 3T3 host cells [81]. Herein we found that 3T3 activity was close to that of *T. cruzi* activity, although higher doses would be needed to confirm the selectivity. We have previously identified this as active against *T. cruzi* from the Pathogen Box collection of compounds (Duffy et al., 2017). In this study, an IC_50_ value could also not be determined against host cells.

Emetine dihydrochloride (**10**) was found to have activity against both *T. b. brucei* and *T. cruzi* with an IC_50_ of 0.05 ± 0.01 and 0.09 ± 0.01 µM, respectively. This compound has shown no selectivity for HEK-293 cells in relation to *T. b. brucei* and *T. cruzi* but has shown moderate selectivity for *T. cruzi* in relation to 3T3 cells, indicating increased cytotoxicity against replicating cells. This compound was previously used as an antiprotozoal drug for the treatment of amoebic liver abscess and invasive intestinal amoebiasis [82]. Its use was discontinued due to severe adverse effects associated with the drug which included cardiotoxic effects [83], severe irritation of oral mucosa, nausea and vomiting [84]. Emetine dihydrochloride has been tested for activity against *T. b. brucei* [85], in which the IC_50_ value was 7.9 µM, however it also displayed poor selectivity (3.8) toward MRC5 cells. No data is currently reported in the literature for activity of this compound against *T. cruzi*.

Chelerythrine chloride (**15**) exhibited an IC_50_ of 0.23 ± 0.04 µM against *T. b. brucei* with good selectivity of >18 fold for HEK-293 cells. Chelerythrine was previously discovered following testing as an oncology drug candidate [86]. Trypanocidal activity has been previously been reported, with an IC_50_ of 1.3 µM against *T. b. brucei* [87].

## 4. Materials and Methods

### 4.1. Maintenance of Mammalian Cells Lines

THP-1 (human monocytic leukemia) cells obtained from American Type Culture Collection (ATCC^®^, Manassas, VA, USA) (THP-1 TIB-202™) were maintained in RPMI (1X) Roswell Park Memorial Institute Medium 1640 GlutaMAX™ (Gibco^®^-Life Technologies, Camarillo, CA, USA) and 10% (*v*/*v*) heat inactivated fetal bovine serum (HIFBS) (Hyclone™ ThermoFisher, Melbourne, Australia) at 37 °C in a humidified atmosphere with 95% air and 5% CO_2_. The cells were passaged every 48 hours to maintain cell density between 2.5 × 10^5^ and 8 × 10^5^ cells/mL.

3T3 host cells (mouse embryo fibroblast; ATCC^®^ CCL92) were maintained in RPMI (Life Technologies, Camarillo ,CA, USA) with no phenol red, supplemented with 10% HIFBS (growth medium).

HEK (human embryonic kidney) 293 cells obtained from the ATCC^®^ (HEK-293 CRL-1573™) were maintained in DMEM (Life Technologies, Camarillo, CA, USA) supplemented with 10% (*v*/*v*) HIFBS at 37 °C in a humidified atmosphere of 95% air and 5% CO_2_. The cells were passaged as they reach 80% confluence.

### 4.2. Maintainanace of Parasites

*L. donovani* (MHOM/IN/80 DD8) promastigotes obtained from ATCC^®^, USA (ATCC^®^ 50212™) were cultivated at 27 °C in Medium 199 (1X) (Gibco^®^-Life Technologies) supplemented with 100 µM adenosine (CAS Number 58-61-7) (Sigma-Aldrich, Castle Hill, Australia), 23 mM folic acid (CAS Number 59-30-3) (Sigma-Aldrich, Castle Hillcity, Australia), 2 mM L-glutamine 200 mM (Gibco^®^-Life Technologies) and 1% (*v*/*v*) BME vitamin mix 100X (Sigma-Aldrich) and 10% (*v*/*v*) HIFBS. The pH of the media was adjusted to 6.8 with 1 M hydrochloric acid (HCl) before being filter sterilised with a cellulose acetate membrane filter, pore size 0.22 µM (Corning^®^ bottle–top vacuum filter system, Oneonta, AL, USA).

*T. b. brucei* bloodstream form strain 427 (BS427), a surrogate model for human infective species commonly used for the determination of compound activity, was kindly supplied by Dr. Achim Schnaufer, (Institute of Cell Biology, University of Edinburgh, UK) whilst at the Seattle Biomedical Research Institute (Seattle, WA, USA) and was cultivated in HMI-9 media at 37 °C in a humidified atmosphere with 95% air and 5% CO_2,_ according to previosuly published protocols [38,88].

*T. cruzi* Tulahuen epimastigotes were generously provided by Professor Frederick Buckner (University of Washington, Seattle, WA, USA). The parasites were maintained in liver infusion tryptose (LIT), with preparation as described by Sykes and Avery [40]. Cells were sub-cultured every 3 or 4 days by diluting 1.25–2.5 × 10^6^ cells/mL respectively in to 10 mLof media in 25 cm^2^ flasks. Cells were grown at 27 °C. Parasites were differentiated into infective trypomastigotes in liquid medium before sub-culturing in 3T3 host cells, as described by Sykes and Avery [40].

### 4.3. Open Access Compound Library and Assay Plate Preparation

The Davis open access natural product-based library currently consists of 472 distinct compounds, the majority (53%) of which are natural products that have been obtained from Australian natural sources, such as endophytic fungi [89], plants [90], macrofungi [91], and marine invertebrates [92]. Approximately 28% of this library contains semi-synthetic natural product analogues [93], while a smaller percentage (19%) are known commercial drugs or synthetic compounds inspired by natural products.

The Davis open access natural product-based library housed within Compounds Australia (www.compoundsaustralia.com), was dispensed into microtitre plates as 5 mM DMSO solutions. Library compounds were either isolated in quantities ranging from 0.2 mg to >50 mg or purchased from commercial suppliers. The natural product isolation procedures or semi-synthetic studies for the majority of compounds in this unique library have been previously published [89,90,91,92]. All compounds were >95% pure when submitted for storage within Compounds Australia.

### 4.4. Phenotypic Assays

For the primary screen, compounds were prepared as a stock concentration of 5 mM in 100% DMSO and tested in duplicate points for two independent experiments (*n* = 2) at final assay concentrations of 20 µM and 16.7 µM for the *L. donovani* intracellular amastigote and promastigote viability assays, respectively. Primary screening against *T. b. brucei* was undertaken at 5 µM and for *T. cruzi* at 10 µM final concentrations. Actives were defined as compounds which exhibited >70% activity at 20 µM and 16.7 µM for *L. donovani* DD8 intracellular amastigote and promastigote assays, respectively. Compounds exhibiting >50% activity at 5 µM for *T. b. brucei* and showing ≥50% activity at a concentration of 10 µM for *T. cruzi* intracellular amastigote were considered active. These compounds were then retested in a 14 point format to determine the IC_50_ value against the parasite, and against HEK-293, THP-1 and 3T3 cells to determine the selectivity to the parasite. To determine IC_50_ values, 14 point dose response curves with top concentrations of 20 µM and 16.7 µM were used for the *L. donovani* DD8 intracellular amastigote and promastigote viability assays, respectively. A top dose of 20 µM was used in the *T. b. brucei* assay and 18.3 µM for *T.cruzi* assay*,* in 14 point dose repsonse curves as described.

#### 4.4.1. *L. donovani* DD8 Intracellular Amastigote Assay

A high content, high-throughput imaging assay was used to identify compounds active against *L. donovani* DD8 intracellular amastigotes as described previosuly [88].

#### 4.4.2. *L. donovani* DD8 Promastigote Viability Assay

This assay was established to assess the activity of the compound library on the extracellular form of the parasites. An initial cell density of 1 × 10^5^ parasites/mL was inoculated into a 75 cm^2^ flask (in a total of 30 mL of M199 medium + 10% (*v*/*v*) HIFBS) and incubated at 27 °C. After 96 h the parasites reached the mid-log phase and were seeded in black, clear bottom 384-well plates (Greiner-bio-one^®^, Monroe, UT, USA) at a concentration of 5 × 10^5^ parasites/mL in a volume of 55 µL using a Bravo automated liquid handling platform (Agilent, Santa Clara, CA, USA). Compound stocks were diluted 1:25 in M199 media without HIFBS using a dilution plate (Greiner-bio-one^®^, Monroe, UT, USA). The compounds were dispensed in 5 µL using a Bravo and parasites incubated for an additional 68 h at 27 °C. Resazurin (Cayman Chemicals^®^, Ann Arbor, MI, USA) at 0.142 mM final assay concentration was then dispensed into plates at a volume of 10 µL/well using a Multidrop™ 384 Reagent Dispenser (Thermo Scientific^®^, Newington, NH, USA) and incubated at 27 °C. The plates were read after 4 h on an EnVision™ Multilabel plate reader (PerkinElmer, Waltham, MA, USA) using fluorometry with an excitation of 530 nm and emission 590 nm. For the *L. donovani* DD8 promastigote viability assay, 1 µM of amphotericin B was used as positive control and 0.4% DMSO as negative control.

#### 4.4.3. *T. b. brucei* Resazurin Viability Assay

The efficacy of compounds against *T. b. brucei* bloodstream form was evaluated using a resazurin-based assay, previously described in the literature [88].

#### 4.4.4. *T. cruzi* Intracellular Amastigote Assay

The efficacy of compounds against *T. cruzi was* undertaken using a phenotypic, high-throughput image-based assay as previously described [40].

#### 4.4.5. HEK-293 Resazurin Viability Assay

Cytotoxicity testing was undertaken as previously described [88].

## 5. Conclusions

The present study identifies several compounds with novel anti-kinetoplastid activities from phenotypic screening of 472 distinct compounds from the Davis open access natural product-based library. Compound **1** displayed in vitro activity against, *L. donovani* DD8, *T. b. brucei* and *T. cruzi* with good selectivity against *T. b. brucei*. Further exploration of the activity of analogues of this compound is warranted to improve anti-parasitic selectivity. Target identification for the most promising compounds will support the future development of pan-active treatments against kinetoplastids. Further compounds from this collection have potential as new chemical starting points for drug discovery efforts against one or more of the parasites tested.

## Figures and Tables

**Table 1:** Dose response and mechanism of action of reference compounds against *L. donovani DD8*, *T. b. brucei* and *T. cruzi*.

Compound	*L. donovani* DD8 Promastigotes	*L. donovani* DD8 Intracellular Amastigotes	*T. b. brucei*	*T. cruzi*	Mechanism of Action
	IC_50_ (mean ± SD) (μM) (S.I HEK-293) (S.I THP-1)	IC_50_ (mean ± SD) (μM) (S.I HEK-293) (S.I THP-1)	IC_50_ (mean ± SD) (μM) (S.I HEK-293)	IC_50_ (mean ± SD) (μM) (S.I HEK-293)	
Amphotericin B	0.12 ± 0.01 (>68.27) ^a^ (>16.39) ^b^	0.20 ± 0.02 (>41.65) ^a^ (>9.61) ^b^	-	-	Binds to ergosterol, the principal sterol in fungal cell membranes and *Leishmania* cells [41].
Miltefosine	3.48 ± 0.26 (>9.57) ^a^ (>5.74) ^b^	2.54 ± 0.57 (>13.12) ^a^ (>7.27) ^b^	-	-	Interacts with lipids (phospholipids and sterols), including membrane lipids, inhibition of cytochrome C oxidase (mitochondrial function), and apoptosis [42,43].
Pentamidine	-	-	0.002 ± 0.001 (>334.52) ^a^	-	Accumulates in trypanosomes; disrupts mitochondrial processes [44].
Diminazene	-	-	0.04 ± 0.01 (>962.75) ^a^	-	Interferes with RNA editing and trans-splicing [44].
Puromycin	-	-	0.03 ± 0.00 (12.96) ^a^	1.65 ± 0.35 (0.150) ^a^	Protein synthesis inhibitor via premature chain termination during translation in the ribosome [44].
Benznidazole	-	-	-	3.36 ± 1.52 (>44.84) ^a^	Causes oxidation in the nucleotide pool which in turn causes the formation of breaks in double stranded DNA [45].
Nifurtimox	-	-	-	0.62 ± 0.10 (> 243.03) ^a^	Unknown. Possibly appears to be due to oxidative stress—potentially from the formation of hydrogen peroxide [46].

All results from two independent experiments (*n* = 2). ^a^ Selectivity index (SI) compared to HEK-293 cells. ^b^ Selectivity index (SI) compared to THP-1 cells.

**Table 2:** In vitro anti-kinetoplastid activities of compounds.

Compounds	Anti-Leishmanial Activity		Anti-Trypanosomal Activity		Comments
	*L. donovani* DD8 Promastigotes	*L. donovani* DD8 Intracellular Amastigotes	*T. b. brucei*	*T. cruzi*	
	IC_50_ (mean ± SD) (μM)	IC_50_ (mean ± SD) (μM)	IC_50_ (mean ± SD) (μM)	IC_50_ (mean ± SD) (μM)	
Lissoclinotoxin E (**1**)	0.72 ± 0.16 (>11.72) ^a^ (6.88) ^b^	4.41 ± 0.24 (>2.26) ^a^ (1.13) ^b^	0.57 ± 0.20 (>17.68) ^a^	3.92 ± 0.38 (>2.52) ^a^(>4.67) ^c^	Active on all three parasites with IC_50_ < 5 uM_._ Exhibits high selectivity for *T. b. brucei* in relation to HEK-293 cells.
Spermatinamine (**2**)	11.87 ± 0.56 (0.33) ^a^ (0.41) ^b^	6.15 ± 0.05 (0.64) ^a^ (0.80) ^b^	1.00 ± 0.26 (5.89) ^a^	-	Active on *L. donovani* DD8 and *T. b. brucei*. Moderate selectivity for *T. b. brucei* in relation to HEK-293 cells.
Thiaplakortone A (**3**)	-	-	3.94 ± 0.78 (0.70) ^a^	4.26 ± 0.65 (0.53) ^a^ (1.06) ^c^	Active on *T. b. brucei* and *T. cruzi* with no selectivity.
Gambogic acid (**4**)	100% inhibition at 16.7 µM	-	0.27 ± 0.04 (7.11) ^a^	1.87 ± 0.07 (1.50) ^a^ (1.00) ^c^	Active on *T .b. brucei* and T. cruzi. Moderately selective for *T. b. brucei* in relation to HEK 293 cells.
Mycophenolic acid (**5**)	-	-	0.51 ± 0.10 (1.01) ^a^	1.59 ± 0.03 (0.37) ^a^ (0.13) ^c^	Active on *T. b. brucei* and *T. cruzi* with no selectivity.
Narciclasine (**6**)	100% inhibition at 16.7 µM	-	0.03 ± 0.01 (1.09) ^a^	0.20 ± 0.01 (0.21) ^a^ (3.28) ^c^	Active on *T. b. brucei* and *T. cruzi*. Low selectivity for *T. cruzi* in 3T3 cells.
Thiaplakortone analogue (**7**)	-	-	0.68 ± 0.01 (4.19) ^a^	3.55 ± 0.38 (1.46) ^a^ (1.33) ^c^	Active on *T. b. brucei* and *T. cruzi*. Moderately selective for *T. b. brucei* in relation to HEK-293 cells.
Mefloquine HCl (**8**)	100% inhibition at 16.7 µM	-	0.62 ± 0.06 (7.97) ^a^	3.96 ± 0.58 (>5.06) ^a^ (>1.70) ^c^	Moderate selectivity for *T. cruzi* and *T. b. brucei* in relation to HEK 293 cells.
3,4-Dihydro-2*H*-naphtho[2,3-*b*]-[1,4] thiazine-5,10-dione 1,1-dioxide (**9**)	100% inhibition at 16.7 µM	-	-	3.81 ± 0.60 (1.11) ^a^ (1.24) ^c^	Active on *L. donovani* DD8 promastigotes and *T. cruzi* with no selectivity.
Emetine dihydrochloride (**10**)	100% inhibition at 16.7 µM	-	0.05 ± 0.01 (1.61) ^a^	0.09 ± 0.00 (0.92) ^a^ (6.04) ^c^	Active on *T. b. brucei* and *T. cruzi* with selectivity for *T. cruzi* in 3T3 cells.
Lissoclinotoxin F (**11**)	5.51 ± 0.30 (>3.62) ^a^ (>1.81) ^b^	8.31 ± 0.67 (>2.40) ^a^ (>1.20) ^b^	-	-	Active on both forms of *L. donovani* DD8 parasite. Low selectivity for both forms of *L. donovani* DD8 parasite.
Ethyl 4-((diethyl-amino)methyl)-5-hydroxy-1-(4-methoxyphenyl)-2-methyl-1*H*-indole-3-carboxylate (**12**)	100% inhibition at 16.7 µM	11.09 ± 0.31 (>2.97) ^a^ (>1.50) ^b^	-	-	Low selectivity for *L. donovani* DD8 intracellular amastigotes.
Chalcone analogue (**13**)	100% inhibition at 16.7 µM	5.65 ± 0.26 (11.87) ^a^ (3.59) ^b^	-	-	Active on *L. donovani* DD8 intracellular amastigotes. Highly selective for *L. donovani* DD8 intracellular amastigotes in relation to HEK 293 cells.
Psammaplysin F (**14**)	-	-	-	5.63 ± 0.76 (>3.51) ^a^ (>1.90) ^c^	Moderate Selectivity for *T. cruzi* in relation to HEK-293 cells.
Chelerythrine chloride (**15**)	-	-	0.23 ± 0.04 (>18.71) ^a^	-	Only selective on *T. b. brucei*. Highly selective for *T. b. brucei* in relation to HEK-293 cells.

All results from two independent experiments (*n* = 2). ^a^ Selectivity index (SI) compared to HEK-293 cells. ^b^ Selectivity index (SI) compared to THP-1 cells. ^c^ Selectivity index (SI) compared to 3T3 cells.

## References

[1] World Health Organization (WHO) (2015). Investing to Overcome the Global Impact of Neglected Tropical Diseases: Third Who Report on negLected Tropical Diseases 2015.

[2] Molyneux D.H., Savioli L., Engel D. (2017). Neglected tropical diseases: Progress towards addressing the chronic pandemic. Lancet.

[3] Borghi S.M., Fattori V., Conchon-Costa I., Pinge-Filho P., Pavanelli W.R., Verri W.A. (2017). Leishmania infection: Painful or painless?. Parasitol. Res..

[4] Herwaldt B.L. (1999). Leishmaniasis. Lancet.

[5] Van Den Abbeele J., Claes Y., Van Bockstaele D., Le Ray D., Coosemans M. (1999). *Trypanosoma brucei* spp. Development in the tsetse fly: Characterization of the post-mesocyclic stages in the foregut and proboscis. Parasitology.

[6] Garcia E., Azambuja P. (1991). Development and interactions of *Trypanosoma cruzi* within the insect vector. Parasitol. Today.

[7] Handman E., Bullen D.V. (2002). Interaction of *Leishmania* with the host macrophage. Trends Parasitol..

[8] Alvar J., Velez I.D., Bern C., Herrero M., Desjeux P., Cano J., Jannin J., den Boer M. (2012). Leishmaniasis worldwide and global estimates of its incidence. PLoS ONE.

[9] Keating J., Yukich J.O., Sutherland C.S., Woods G., Tediosi F. (2015). Human african trypanosomiasis prevention, treatment and control costs: A systematic review. Acta Trop..

[10] Bonney K.M., Engman D.M. (2015). Autoimmune pathogenesis of chagas heart disease: Looking back, looking ahead. Am. J. Pathol..

[11] McCall L.-I., McKerrow J.H. (2014). Determinants of disease phenotype in trypanosomatid parasites. Trends Parasitol..

[12] Sundar S., Chakravarty J. (2015). An update on pharmacotherapy for leishmaniasis. Expert Opin. Pharmacother..

[13] Jones A.J., Avery V.M. (2015). Future treatment options for human african trypanosomiasis. Expert Rev. Anti-Infect. Ther..

[14] Bermudez J., Davies C., Simonazzi A., Real J.P., Palma S. (2016). Current drug therapy and pharmaceutical challenges for chagas disease. Acta Trop..

[15] Mondelaers A., Sanchez-Cañete M.P., Hendrickx S., Eberhardt E., Garcia-Hernandez R., Lachaud L., Cotton J., Sanders M., Cuypers B., Imamura H. (2016). Genomic and molecular characterization of miltefosine resistance in *Leishmania infantum* strains with either natural or acquired resistance through experimental selection of intracellular amastigotes. PLoS ONE.

[16] Barrett M.P., Vincent I.M., Burchmore R.J., Kazibwe A.J., Matovu E. (2011). Drug resistance in human african trypanosomiasis. Future Med..

[17] Campos M.C.O., Leon L.L., Taylor M.C., Kelly J.M. (2014). Benznidazole-resistance in *trypanosoma cruzi*: Evidence that distinct mechanisms can act in concert. Mol. Biochem. Parasitol..

[18] Don R., Ioset J.R. (2014). Screening strategies to identify new chemical diversity for drug development to treat kinetoplastid infections. Parasitology.

[19] Brun R., Blum J., Chappuis F., Burri C. (2010). Human african trypanosomiasis. Lancet.

[20] Aronson N.E. (2017). Addressing a clinical challenge: Guidelines for the diagnosis and treatment of leishmaniasis. BMC Med..

[21] Bhuniya D., Mukkavilli R., Shivahare R., Launay D., Dere R.T., Deshpande A., Verma A., Vishwakarma P., Moger M., Pradhan A. (2015). Aminothiazoles: Hit to lead development to identify antileishmanial agents. Eur. J. Med. Chem..

[22] Mowbray C.E., Braillard S., Speed W., Glossop P.A., Whitlock G.A., Gibson K.R., Mills J.E., Brown A.D., Gardner J.M., Cao Y. (2015). Novel amino-pyrazole ureas with potent in vitro and in vivo antileishmanial activity. J. Med. Chem..

[23] Zulfiqar B., Shelper T.B., Avery V.M. (2017). Leishmaniasis drug discovery: Recent progress and challenges in assay development. Drug Discov. Today.

[24] Torreele E., Trunz B.B., Tweats D., Kaiser M., Brun R., Mazue G., Bray M.A., Pecoul B. (2010). Fexinidazole—A new oral nitroimidazole drug candidate entering clinical development for the treatment of sleeping sickness. PLoS Negl. Trop. Dis..

[25] Jacobs R.T., Nare B., Wring S.A., Orr M.D., Chen D., Sligar J.M., Jenks M.X., Noe R.A., Bowling T.S., Mercer L.T. (2011). Scyx-7158, an orally-active benzoxaborole for the treatment of stage 2 human african trypanosomiasis. PLoS Negl. Trop. Dis..

[26] Kaiser M., Bray M.A., Cal M., Bourdin Trunz B., Torreele E., Brun R. (2011). Antitrypanosomal activity of fexinidazole, a new oral nitroimidazole drug candidate for treatment of sleeping sickness. Antimicrob. Agents Chemother..

[27] Field M.C., Horn D., Fairlamb A.H., Ferguson M.A., Gray D.W., Read K.D., De Rycker M., Torrie L.S., Wyatt P.G., Wyllie S. (2017). Anti-trypanosomatid drug discovery: An ongoing challenge and a continuing need. Nat. Rev. Microbiol..

[28] Kola I., Landis J. (2004). Can the pharmaceutical industry reduce attrition rates?. Nat. Rev. Drug Discov..

[29] Nwaka S., Besson D., Ramirez B., Maes L., Matheeussen A., Bickle Q., Mansour N.R., Yousif F., Townson S., Gokool S. (2011). Integrated dataset of screening hits against multiple neglected disease pathogens. PLoS Negl. Trop. Dis..

[30] Silva J.R., Ramos Ade S., Machado M., de Moura D.F., Neto Z., Canto-Cavalheiro M.M., Figueiredo P., do Rosario V.E., Amaral A.C., Lopes D. (2011). A review of antimalarial plants used in traditional medicine in communities in portuguese-speaking countries: Brazil, mozambique, cape verde, guinea-bissau, sao tome and principe and angola. Mem. Inst. Oswaldo Cruz.

[31] Althaus J.B., Malyszek C., Kaiser M., Brun R., Schmidt T.J. (2017). Alkamides from *Anacyclus pyrethrum* l. and their in vitro antiprotozoal activity. Molecules.

[32] Yang X., Feng Y., Duffy S., Avery V.M., Camp D., Quinn R.J., Davis R.A. (2011). A new quinoline epoxide from the australian plant *Drummondita calida*. Planta Med..

[33] Davis R.A., Sykes M., Avery V.M., Camp D., Quinn R.J. (2011). Convolutamines I and J, antitrypanosomal alkaloids from the bryozoan *Amathia tortusa*. Bioorg. Med. Chem..

[34] Feng Y., Davis R.A., Sykes M.L., Avery V.M., Quinn R.J. (2012). Iotrochamides A and B, antitrypanosomal compounds from the Australian marine sponge *Iotrochota* sp.. Bioorg. Med. Chem. Lett..

[35] Davis R.A., Duffy S., Fletcher S., Avery V.M., Quinn R.J. (2013). Thiaplakortones A-D: Antimalarial thiazine alkaloids from the Australian marine sponge *Plakortis lita*. J. Org. Chem..

[36] Choomuenwai V., Beattie K.D., Healy P.C., Andrews K.T., Fechner N., Davis R.A. (2015). Entonalactams A–C: Isoindolinone derivatives from an Australian rainforest fungus belonging to the genus *Entonaema*. Phytochemistry.

[37] Siqueira-Neto J.L., Moon S., Jang J., Yang G., Lee C., Moon H.K., Chatelain E., Genovesio A., Cechetto J., Freitas-Junior L.H. (2012). An image-based high-content screening assay for compounds targeting intracellular *Leishmania donovani* amastigotes in human macrophages. PLoS Negl. Trop. Dis..

[38] Sykes M.L., Avery V.M. (2009). Development of an alamar blue viability assay in 384-well format for high throughput whole cell screening of *Trypanosoma brucei brucei* bloodstream form strain 427. Am. J. Trop. Med. Hyg..

[39] Jones A.J., Kaiser M., Avery V.M. (2015). Identification and characterization of fty720 for the treatment of human african trypanosomiasis. Antimicrob. Agents Chemother..

[40] Sykes M.L., Avery V.M. (2015). Development and application of a sensitive, phenotypic, high-throughput image-based assay to identify compound activity against *Trypanosoma cruzi* amastigotes. Int. J. Parasitol. Drugs Drug Resist..

[41] Roberts C., McLeod R., Rice D., Ginger M., Chance M.L., Goad L.J. (2003). Fatty acid and sterol metabolism: Potential antimicrobial targets in apicomplexan and trypanosomatid parasitic protozoa. Mol. Biochem. Parasitol..

[42] Freitas-Junior L.H., Chatelain E., Kim H.A., Siqueira-Neto J.L. (2012). Visceral leishmaniasis treatment: What do we have, what do we need and how to deliver it?. Int. J. Parasitol. Drugs Drug Resist..

[43] Luque-Ortega J.R., Rivas L. (2007). Miltefosine (hexadecylphosphocholine) inhibits cytochrome c oxidase in *Leishmania donovani* promastigotes. Antimicrob. Agents Chemother..

[44] Barrett M.P., Boykin D.W., Brun R., Tidwell R.R. (2007). Human african trypanosomiasis: Pharmacological re-engagement with a neglected disease. Br. J. Pharmacol..

[45] Rajao M.A., Furtado C., Alves C.L., Passos-Silva D.G., de Moura M.B., Schamber-Reis B.L., Kunrath-Lima M., Zuma A.A., Vieira-da-Rocha J.P., Garcia J.B.F. (2014). Unveiling benznidazole’s mechanism of action through overexpression of DNA repair proteins in *Trypanosoma cruzi*. Environ. Mol. Mutagen..

[46] Boiani M., Piacenza L., Hernández P., Boiani L., Cerecetto H., González M., Denicola A. (2010). Mode of action of nifurtimox and n-oxide-containing heterocycles against *Trypanosoma cruzi*: Is oxidative stress involved?. Biochem. Pharmacol..

[47] Annang F., Pérez-Moreno G., García-Hernández R., Cordon-Obras C., Martín J., Tormo J., Rodríguez L., De Pedro N., Gómez-Pérez V., Valente M. (2015). High-throughput screening platform for natural product–based drug discovery against 3 neglected tropical diseases: Human african trypanosomiasis, leishmaniasis, and chagas disease. J. Biomol. Screen..

[48] Jones A.J., Grkovic T., Sykes M.L., Avery V.M. (2013). Trypanocidal activity of marine natural products. Mar. Drugs.

[49] Stuart K., Brun R., Croft S., Fairlamb A., Gurtler R.E., McKerrow J., Reed S., Tarleton R. (2008). Kinetoplastids: Related protozoan pathogens, different diseases. J. Clin. Investig..

[50] Baiocco P., Poce G., Alfonso S., Cocozza M., Porretta G.C., Colotti G., Biava M., Moraca F., Botta M., Yardley V. (2013). Inhibition of *Leishmania infantum* trypanothione reductase by azole-based compounds: A comparative analysis with its physiological substrate by x-ray crystallography. ChemMedChem.

[51] Corona P., Gibellini F., Cavalli A., Saxena P., Carta A., Loriga M., Luciani R., Paglietti G., Guerrieri D., Nerini E. (2012). Structure-based selectivity optimization of piperidine-pteridine derivatives as potent *Leishmania* pteridine reductase inhibitors. J. Med. Chem..

[52] Schroder J., Noack S., Marhofer R.J., Mottram J.C., Coombs G.H., Selzer P.M. (2013). Identification of semicarbazones, thiosemicarbazones and triazine nitriles as inhibitors of *Leishmania mexicana* cysteine protease cpb. PLoS ONE.

[53] Cavalli A., Lizzi F., Bongarzone S., Brun R., Luise Krauth-Siegel R., Bolognesi M.L. (2009). Privileged structure-guided synthesis of quinazoline derivatives as inhibitors of trypanothione reductase. Bioorg. Med. Chem. Lett..

[54] Mpamhanga C.P., Spinks D., Tulloch L.B., Shanks E.J., Robinson D.A., Collie I.T., Fairlamb A.H., Wyatt P.G., Frearson J.A., Hunter W.N. (2009). One scaffold, three binding modes: Novel and selective pteridine reductase 1 inhibitors derived from fragment hits discovered by virtual screening. J. Med. Chem..

[55] Breuning A., Degel B., Schulz F., Buchold C., Stempka M., Machon U., Heppner S., Gelhaus C., Leippe M., Leyh M. (2010). Michael acceptor based antiplasmodial and antitrypanosomal cysteine protease inhibitors with unusual amino acids. J. Med. Chem..

[56] DND*i* Fexinidazole (hat).

[57] Wyllie S., Patterson S., Stojanovski L., Simeons F.R., Norval S., Kime R., Read K.D., Fairlamb A.H. (2012). The anti-trypanosome drug fexinidazole shows potential for treating visceral leishmaniasis. Sci. Transl. Med..

[58] Marr J., Muller M. (1995). Biochemistry and Molecular Biology of Parasites.

[59] Davis R.A., Sandoval I.T., Concepcion G.P., da Rocha R.M., Ireland C.M. (2003). Lissoclinotoxins e and f, novel cytotoxic alkaloids from a Philippine *Didemnid ascidian*. Tetrahedron.

[60] Buchanan M.S., Carroll A.R., Fechner G.A., Boyle A., Simpson M.M., Addepalli R., Avery V.M., Hooper J.N., Su N., Chen H. (2007). Spermatinamine, the first natural product inhibitor of isoprenylcysteine carboxyl methyltransferase, a new cancer target. Bioorg. Med. Chem. Lett..

[61] Yin S., Davis R.A., Shelper T., Sykes M.L., Avery V.M., Elofsson M., Sundin C., Quinn R.J. (2011). Pseudoceramines A–D, new antibacterial bromotyrosine alkaloids from the marine sponge *Pseudoceratina* sp.. Org. Biomol. Chem..

[62] Choomuenwai V., Schwartz B.D., Beattie K.D., Andrews K.T., Khokhar S., Davis R.A. (2013). The discovery, synthesis and antimalarial evaluation of natural product-based polyamine alkaloids. Tetrahedron Lett..

[63] Buckner F.S., Kateete D.P., Lubega G.W., Van Voorhis W.C., Yokoyama K. (2002). *Trypanosoma brucei* prenylated-protein carboxyl methyltransferase prefers farnesylated substrates. Biochem. J..

[64] Schwartz B.D., Coster M.J., Skinner-Adams T.S., Andrews K.T., White J.M., Davis R.A. (2015). Synthesis and antiplasmodial evaluation of analogues based on the tricyclic core of thiaplakortones A-D. Mar. Drugs.

[65] Schwartz B.D., Skinner-Adams T.S., Andrews K.T., Coster M.J., Edstein M.D., MacKenzie D., Charman S.A., Koltun M., Blundell S., Campbell A. (2015). Synthesis and antimalarial evaluation of amide and urea derivatives based on the thiaplakortone a natural product scaffold. Org. Biomol. Chem..

[66] Zhang H.-Z., Kasibhatla S., Wang Y., Herich J., Guastella J., Tseng B., Drewe J., Cai S.X. (2004). Discovery, characterization and sar of gambogic acid as a potent apoptosis inducer by a hts assay. Bioorg. Med. Chem..

[67] Mackey Z.B., Baca A.M., Mallari J.P., Apsel B., Shelat A., Hansell E.J., Chiang P.K., Wolff B., Guy K.R., Williams J. (2006). Discovery of trypanocidal compounds by whole cell hts of *Trypanosoma brucei*. Chem. Biol. Drug Des..

[68] Wu X., Cao S., Goh S., Hsu A., Tan B.K. (2002). Mitochondrial destabilisation and caspase-3 activation are involved in the apoptosis of jurkat cells induced by gaudichaudione a, a cytotoxic xanthone. Planta Med..

[69] Bessho T., Morii S., Kusumoto T., Shinohara T., Noda M., Uchiyama S., Shuto S., Nishimura S., Djikeng A., Duszenko M. (2013). Characterization of the novel *Trypanosoma brucei* inosine 5′-monophosphate dehydrogenase. Parasitology.

[70] Cao S., Aboge G.O., Terkawi M.A., Zhou M., Luo Y., Yu L., Li Y., Goo Y., Kamyingkird K., Masatani T. (2013). Cloning, characterization and validation of inosine 5′-monophosphate dehydrogenase of *Babesia gibsoni* as molecular drug target. Parasitol. Int..

[71] Sullivan W.J., Dixon S.E., Li C., Striepen B., Queener S.F. (2005). Imp dehydrogenase from the protozoan parasite *Toxoplasma gondii*. Antimicrob. Agents Chemother..

[72] Suganuma K., Sarwono A.E., Mitsuhashi S., Jakalski M., Okada T., Nthatisi M., Yamagishi J., Ubukata M., Inoue N. (2016). Mycophenolic acid and its derivatives as potential chemotherapeutic agents targeting inosine monophosphate dehydrogenase in *Trypanosoma congolense*. Antimicrob. Agents Chemother..

[73] Ceriotti G. (1967). Narciclasine: An antimitotic substance from narcissus bulbs. Nature.

[74] Carrasco L., Fresno M., Vazquez D. (1975). Narciclasine: An antitumour alkaloid which blocks peptide bond formation by eukaryotic ribosomes. FEBS Lett..

[75] Gabrielsen B., Monath T.P., Huggins J.W., Kirsi J.J., Hollingshead M., Shannon W.M., Pettit G.R. (1992). Activity of selected *Amaryllidaceae* constituents and related synthetic substances against medically important RNA viruses. Natural Products as Antiviral Agents.

[76] Yang X., Davis R.A., Buchanan M.S., Duffy S., Avery V.M., Camp D., Quinn R.J. (2010). Antimalarial bromotyrosine derivatives from the australian marine sponge *Hyattella* sp.. J. Nat. Prod..

[77] Salem M.M., Werbovetz K.A. (2005). Antiprotozoal compounds from *Psorothamnus polydenius*. J. Nat. Prod..

[78] Croft S.L., Yardley V., Kendrick H. (2002). Drug sensitivity of *Leishmania species*: Some unresolved problems. Trans. R. Soc. Trop. Med. Hyg..

[79] Neal R., Allen S., McCoy N., Olliaro P., Croft S. (1995). The sensitivity of *Leishmania* species to aminosidine. J. Antimicrob. Chemother..

[80] Otigbuo I.N., Onabanjo A.O. (1992). The in vitro and in vivo effects of mefloquine on *Trypanosoma brucei brucei*. J. Hyg. Epidemiol. Microbiol. Immunol..

[81] Planer J.D., Hulverson M.A., Arif J.A., Ranade R.M., Don R., Buckner F.S. (2014). Synergy testing of fda-approved drugs identifies potent drug combinations against *Trypanosoma cruzi*. PLoS Negl. Trop. Dis..

[82] Lasserre R. (1979). Treatment of amoebiasis. Phil. J. Microbiol. Infect. Dis..

[83] Lemmens-Gruber R., Karkhaneh A., Studenik C., Heistracher P. (1996). Cardiotoxicity of emetine dihydrochloride by calcium channel blockade in isolated preparations and ventricular myocytes of guinea-pig hearts. Br. J. Pharmacol..

[84] Matthews H., Usman-Idris M., Khan F., Read M., Nirmalan N. (2013). Drug repositioning as a route to anti-malarial drug discovery: Preliminary investigation of the in vitro anti-malarial efficacy of emetine dihydrochloride hydrate. Malaria J..

[85] Jones D.C., Hallyburton I., Stojanovski L., Read K.D., Frearson J.A., Fairlamb A.H. (2010). Identification of a kappa-opioid agonist as a potent and selective lead for drug development against human african trypanosomiasis. Biochem. Pharmacol..

[86] Chan S.L., Lee M.C., Tan K.O., Yang L.K., Lee A.S., Flotow H., Fu N.Y., Butler M.S., Soejarto D.D., Buss A.D. (2003). Identification of chelerythrine as an inhibitor of bclxl function. J. Biol. Chem..

[87] Rosenkranz V., Wink M. (2008). Alkaloids induce programmed cell death in bloodstream forms of trypanosomes (*trypanosoma b. Brucei*). Molecules.

[88] Duffy S., Sykes M.L., Jones A.J., Shelper T.B., Simpson M., Lang R., Poulsen S.A., Sleebs B.E., Avery V.M. (2017). Screening the mmv pathogen box across multiple pathogens reclassifies starting points for open source drug discovery. Antimicrob. Agents Chemother..

[89] Davis R.A. (2005). Isolation and structure elucidation of the new fungal metabolite (−)-xylariamide a. J. Nat. Prod..

[90] Levrier C., Balastrier M., Beattie K.D., Carroll A.R., Martin F., Choomuenwai V., Davis R.A. (2013). Pyridocoumarin, aristolactam and aporphine alkaloids from the Australian rainforest plant *Goniothalamus australis*. Phytochemistry.

[91] Choomuenwai V., Andrews K.T., Davis R.A. (2012). Synthesis and antimalarial evaluation of a screening library based on a tetrahydroanthraquinone natural product scaffold. Bioorg. Med. Chem..

[92] Barnes E.C., Said N.A.B., Williams E.D., Hooper J.N., Davis R.A. (2010). Ecionines A and B, two new cytotoxic pyridoacridine alkaloids from the Australian marine sponge, *Ecionemia geodides*. Tetrahedron.

[93] Barnes E.C., Kumar R., Davis R.A. (2016). The use of isolated natural products as scaffolds for the generation of chemically diverse screening libraries for drug discovery. Nat. Prod. Rep..

